# Hypothesis and Theory: Evaluating the Co-Evolution of the Melanocortin-2 Receptor and the Accessory Protein MRAP1

**DOI:** 10.3389/fendo.2021.747843

**Published:** 2021-11-01

**Authors:** Robert M. Dores, Emilia Chapa

**Affiliations:** Department of Biological Sciences, University of Denver, Denver, CO, United States

**Keywords:** melanocortin-2 receptor, MRAP1, evolution, HPA Axis, HPI Axis

## Abstract

The melanocortin receptors (MCRs) and the MRAP accessory proteins belong to distinct gene families that are unique to the chordates. During the radiation of the chordates, the melancortin-2 receptor paralog (MC2R) and the MRAP1 paralog (melanocortin-2 receptor accessory protein 1) have co-evolved to form a heterodimer interaction that can influence the ligand selectivity and trafficking properties of MC2R. This apparently spontaneous interaction may have begun with the ancestral gnathostomes and has persisted in both the cartilaginous fishes and the bony vertebrates. The ramifications of this interaction had profound effects on the hypothalamus/anterior pituitary/adrenal-interrenal axis of bony vertebrates resulting in MC2R orthologs that are exclusively selective for the anterior pituitary hormone, ACTH, and that are dependent on MRAP1 for trafficking to the plasma membrane. The functional motifs within the MRAP1 sequence and their potential contact sites with MC2R are discussed. The ramifications of the MC2R/MRAP1 interaction for cartilaginous fishes are also discussed, but currently the effects of this interaction on the hypothalamus/pituitary/interrenal axis is less clear. The cartilaginous fish MC2R orthologs have apparently retained the ability to be activated by either ACTH or MSH-sized ligands, and the effect of MRAP1 on trafficking varies by species. In this regard, the possible origin of the dichotomy between cartilaginous fish and bony vertebrate MC2R orthologs with respect to ligand selectivity and trafficking properties is discussed in light of the evolution of functional amino acid motifs within MRAP1.

## Introduction

The melanocortin receptors (MCRs) are a family of chordate G protein-coupled receptors (GPCRs), and for most species in this phylum there are five paralogous melanocortin receptor genes [i.e., *mc1r, mc2r, mc3r, mc4r and mc5r;* (1–3)]. These receptors are activated by melanocortin peptides such as, α-melanocyte-stimulating hormone (α-MSH), β-melanocyte-stimulating hormone (β-MSH), γ-melanocyte-stimulating hormone (γ-MSH), and adrenocorticotropin (ACTH) which are all derived from the prohormone precursor protein, proopiomelanocortin (POMC) (4–6).

In terms of function, studies on mammals, as previously reviewed (1), indicate that MC1R is involved in the regulation of pigmentation, body temperature regulation, and modulation of anti-inflammatory mechanisms. This receptor is located on melanocytes in the integument, in various brain regions, and on macrophages, respectively. MC2R is involved in glucocorticoid production, and is expressed in cells of the adrenal cortex, but it also expressed on melanocytes and adipocytes. MC3R and MC4R are involved in appetite regulation and energy balance, and are predominantly, but not exclusively, expressed by neurons in the central nervous system. Lastly, MC5R plays a role in sebaceous gland physiology, and this receptor is expressed in many tissues including the adrenal gland, skin, and various exocrine glands.

## Properties of MC2R and the Discovery of MRAP

While MCRs such as MC1R, MC3R, MC4R, and MC5R, can be activated by any melanocortin peptide, mammalian MC2R orthologs can only be activated by ACTH (1, 7, 8). In addition, MC1R, MC3R, MC4R, and MC5R can be functionally expressed in most mammalian cell lines. However, MC2R can only be functionally expressed in cells derived from the adrenal cortex, but not in the various mammalian cell lines (e.g., CHO cells, COS cells, or HEK-293 cells) usually used to analyze the pharmacological properties of GPCRs (9).

**Figure 1:**
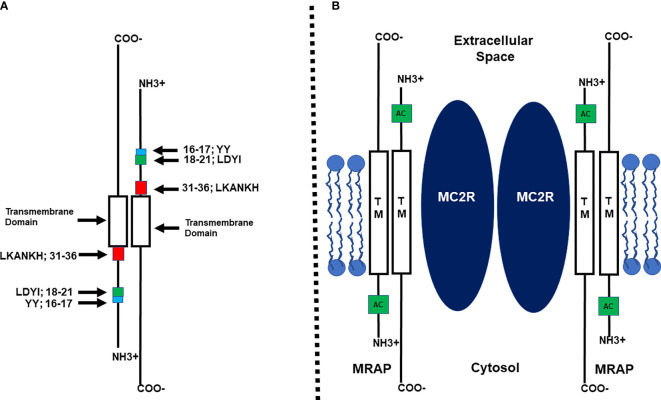
**(A)** Schematic representation of the functional regions of mouse MRAP1 (not drawn to scale) based on the study by Sebag and Hinckle (14). Within the N-terminal domain there are three functional motifs: Y^16^Y^17^, the extended activation motif; L^18^D^19^Y^20^I^21^, the activation motif; L^31^K^32^A^33^N^34^K^35^H^36^, the reverse topology motif. The Transmembrane domain (TM) is required for facilitating the trafficking of MC2R to the plasma membrane. **(B)** Diagram of the MC2R/MRAP1 heterodimer (not drawn to scale) based on the study by Cooray et al. (15). Two MRAP homodimers form a heterodimer with a MC2R homodimer. TM, Transmembrane Domain; AC, Activation motif.

The inability to functionally express MC2R in most mammalian cells lines was resolved when Metherell et al. (10) characterized the accessory protein, MRAP (melanocortin-2 receptor accessory protein). This single pass transmembrane protein is coded on a single gene that contains 6 exons, and the human ortholog does undergo alternative splicing to yield two isoforms: MRAP-α and MRAP-β (10). These isoforms have the same N-terminal domain and transmembrane domain but are divergent in the amino acid sequence of their respective C-terminal domains. Co-transfection of human MRAPα or MRAPβ with human MC2R in any of a number of mammalian cells lines such as CHO or HEK-290 cells indicated that the transfected cells can be stimulated at physiologically relevant doses of human ACTH and the result was a robust production of cAMP by the transfected cells as reviewed by Webb and Clark (11). Hence, the functional activation of adrenal cortex cells resulting in the production and secretion of glucocorticoids is dependent on the formation of a heterodimer between MC2R and MRAP (10). In addition, this study also solved another enigma. Clinical studies had found that only 25% of the cases of Familial Glucocorticoid Deficiency, (FGD; newborn humans unable to produce a functional cortisol response) were due to point mutations in the *MC2R* gene (FGD Type 1). The Metherell et al. (10) study concluded that another cause of FDG can be mutations to the human *MRAP* gene that disrupt the interaction between MC2R and MRAP (i.e., FDG Type 2). Finally, the discovery of MRAP indicated that the formation of the MC2R/MRAP heterodimer was required for both the trafficking of MC2R from the endoplasmic reticulum to the plasma membrane and for the activation of MC2R following the binding of ACTH to the receptor (11, 12). Subsequent structure/function analyses by Sebag and Hinkle (13, 14) using the MRAP ortholog of the mouse as a model system (mMRAP) would reveal several novel features of this accessory protein which are summarized in **Figure 1A** (12).

MRAP has been classified as a class 1 transmembrane protein due to the fact that at the plasma membrane the N-terminal domain of some of the accessory protein monomers do face the extracellular space. However, it became apparent that MRAP monomers form a homodimer with reverse topology [(13); **Figure 1A**]. Deletion mutants of mMRAP indicated that removal of the LKANKH motif in the N-terminus of the accessory protein prevented the reverse topology orientation from forming [(14); **Figure 1A**]. This outcome not only blocked MRAP homodimer formation, but also blocked heterodimer interaction between MC2R and MRAP which interfered with the trafficking and activation of the receptor. With respect to the trafficking function of MRAP, a chimeric protein paradigm revealed that substitution of the transmembrane domain of mMRAP with the corresponding domain from RAMP3, the accessory protein for the CGRP receptor [calcitonin gene-related peptide receptor; (16)] completely blocked trafficking of human MC2R to the Plasma Membrane [(14); **Figure 1A**]. Thus the trafficking of MC2R from the ER to the plasma membrane is dependent on the transmembrane domain of the MRAP homodimer making contact with a transmembrane domain of MC2R.

Another functional amino acid motif was uncovered in the N-terminal domain of mMRAP that did not involve the reverse topology of the MRAP homodimer or trafficking of the MC2R/MRAP heterodimer but did block activation of MC2R at the plasma membrane (14). Using an alanine substitution paradigm, Sebag and Hinkle (14) observed that alanine replacement of the LDYI motif in mMRAP completely blocked activation of MC2R (**Figure 1A**). However, while alanine substitution at the Y residue in the LDYI motif resulted in a 50% decrease in the activation of MC2R, single alanine substitution at the other residues in the motif (i.e., L, D, or I) had no negative effect on activation. These results were perplexing and either point to a role for the secondary structure of the LDYI motif in the activation of MC2R, or perhaps indicate that additional residues near the LDYI motif are needed for facilitating the activation of the receptor. Finally, a bioluminescence resonance energy transfer analysis concluded that at the plasma membrane, the MC2R/MRAP complex is a MC2R homodimer interacting with two MRAP homodimers as depicted in **Figure 1B** (15).

While the discovery of MRAP (10) removed an impasse for analyzing the pharmacology of MC2R, several issues remained unresolved. One of these issues was, since MRAP has reverse topology, and each monomer of the homodimer has an activation motif (i.e., LDYI), does the MRAP activation motif interact with an extracellular domain of MC2R or an intracellular domain of the receptor to facilitate activation of the receptor (**Figure 1B**)? This issue was partially resolved using an ingenious chimeric protein paradigm as described in Malik et al. (17). This study presented convincing evidence that the activation motif on the N-terminal domain of the MRAP monomer facing the extracellular space side of the plasma membrane was interacting with an extracellular domain on MC2R. However, the later study did not identify the specific extracellular domain on MC2R that makes contact with the N-terminal domain of MRAP. In addition, another unresolved issue is the identity of the transmembrane domain(s) on MC2R that interacts with the transmembrane domain of MRAP to facilitate trafficking of the heterodimer.

The Malek et al. (17) study also found that the two Y residues immediately N-terminal to the LDYI motif in mMRAP are needed for full activation of MC2R (**Figure 1A**). This observation suggested that the “activation motif” on the N-terminus of mMRAP includes more residues than just the LYDI motif.

With respect to the identity of the extracellular domain of MC2R that makes contact with the N-terminal activation motif of MRAP, several studies of human (h) MC2R point to Extracellular Loop 2 (EC2). Alanine substitution of F^168^ in the EC2 domain results in a decrease in activation of hMC2R (18). In addition, a patient with FGD Type 1 had a point mutation resulting in an amino acid substitution at H^170^ in the EC2 domain (19) which rendered the patient’s MC2R inactive, but had no apparent effect on the trafficking of the mutant receptor. Support for this conclusion came from an alanine substitution study. When the hMC2R (H^170^ to A^170^) mutant was co-expressed in CHO cells with mMRAP there was a significant decreased in activation of the mutant hMC2R following stimulation with hACTH(1-24) as compared to the positive control (20). In addition, using a chimeric receptor paradigm, Fridmanis et al. (21), implicated EC2 as an important domain for the activation of hMC2R.

To summarize, by 2011 it was clear that the functional expression of mammalian MC2R orthologs was dependent on the formation of a heterodimer between the receptor and the accessory protein, MRAP, that most likely occurred at the endoplasmic reticulum (11, 12). Functional MRAP is a homodimer with reverse topology as a result of the presence of the LKANKH motif in the N-terminus of each MRAP monomer of the MRAP homodimer (13). The efficient movement of the MC2R/MRAP heterodimer from the ER to the Golgi to the plasma membrane is dependent on the transmembrane domain of MRAP presumably making contact with a corresponding transmembrane domain in the receptor. Activation of MC2R at the plasma membrane following the binding of ACTH is at least partially dependent on the LDYI motif in the N-terminal heterodimer of MRAP (**Figure 1**).

## Phylogeny of MRAPs and MCRs in Vertebrates

**Figure 2:**
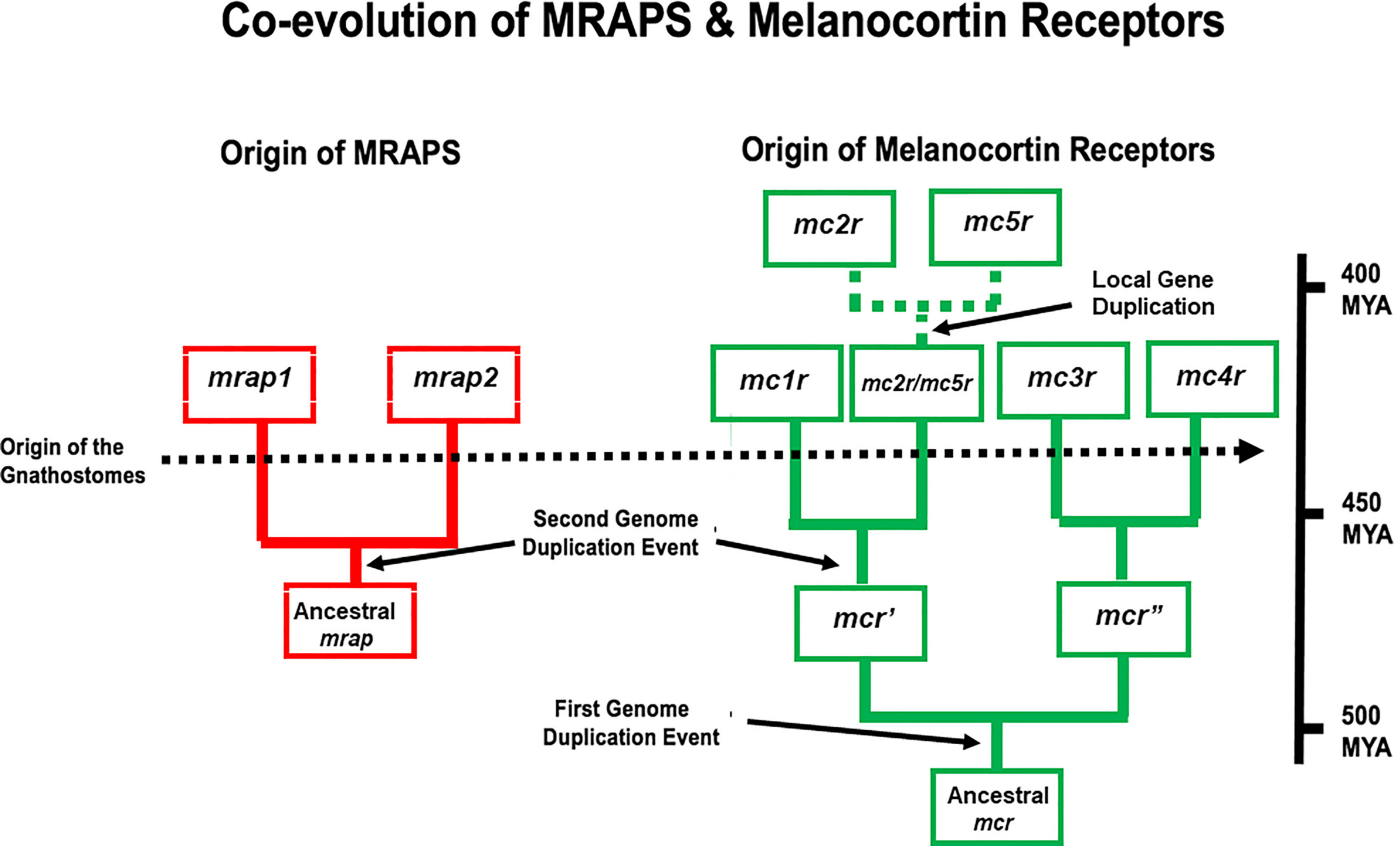
A schematic representation of genome duplication events in the proposed co-evolution of MRAPs and MC2Rs. This figure is modified from (25). In this scenario, the ancestral melanocortin gene (*mcr*) was duplicated as a result of the R1 genome duplication event to give rise to *mcr’* and mcr”. As a result of the R2 genome duplication event, four paralogs emerged *mc1r*, *mc3r, mc4r*, and *mc2r/mc5r*. This scenario proposes that distinct *mc2r* and *mc5r* paralogs were the result of a local gene duplication event that occurred in the ancestral gnathostomes. In this scenario, the ancestral *mrap* gene evolved in R1 agnathan vertebrates and duplicated to yield the *mrap1* and *mrap2* genes as a a result of the R2 genome duplication event. MYA, millions of years.

The novel interaction between MC2R and MRAP is not limited to mammals, and the co-evolution of MC2R and MRAP most likely become intertwined early in the radiation of the gnathostomes. Given the number of melanocortin receptor-coding genes, and the presence of four of the five receptor-coding genes (i.e., *mc1r, mc2r, mc3r*, and *mc4r*) on separate chromosomes in gnathostome genomes, it appears that this gene family was the result of the two predicted genome duplication events that occurred during the early radiation of the chordates (22–24). A hypothetical scheme for the radiation of the melanocortin receptors is presented in **Figure 2**. In addition, the presence of the *mc2r* gene and the *mc5r* gene on the same chromosome in many vertebrate genomes would appear to be the result of a local gene duplication, and this proposed duplication event may have occurred in the ancestral gnathostomes (25).

The *mrap* gene and its paralog the *mrap2* gene (26) form their own gene family, and *mrap2* orthologs have been found in every vertebrate genome that has been annotated (2). For example, hMRAP2 orthologs have a reverse topology motif and high sequence identity in the transmembrane domain with hMRAP but lacks an activation motif in the N-terminal domain (26). With the discovery of hMRAP2 in 2009, it seemed reasonable to begin referring to “MRAP” as “MRAP1”, and this nomenclature will be used for the remainder of this review. In vertebrate genomes, the *mrap1* gene and the *mrap2* gene are located on separate chromosomes and appear to be the result of the second chordate genome duplication event. A scenario for the evolution of the *mrap* gene family is shown in **Figure 2**.

With the realization that mammalian MC2R required co-expression with a mammalian MRAP1 for functional expression in mammalian cell lines, a series of comparative studies began in 2010 to understand the pharmacological properties of bony vertebrate MC2R orthologs. The initial study was done on the MC2R ortholog of a teleost fish (27), followed by subsequent studies on representative non-teleost bony fish, amphibian, reptile, and avian species (28–35). In every study the respective MC2R ortholog required co-expression with an MRAP1 ortholog for activation when expressed in mammalian cell lines such CHO cells or HEK-293 cells. Collectively, these studies indicate that the obligate interaction between MC2R and MRAP1 must have emerged early in the radiation of the bony vertebrates (i.e., bony fishes, amphibians, reptiles, birds, and mammals). In addition, all of the bony vertebrate MC2R orthologs that have been analyzed are exclusively selective for activation by ACTH and display minimal or no activation when stimulated with αMSH.

**Figure 3:**
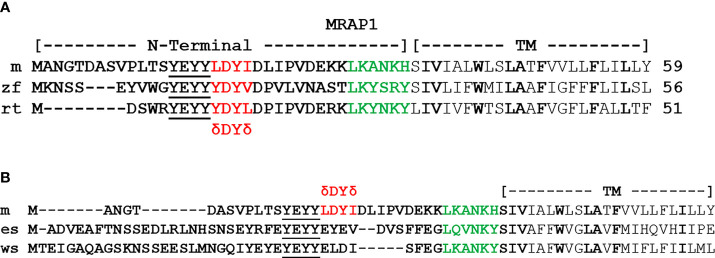
Alignment of bony vertebrate MRAP1sequences. **(A)** The amino acid sequences of the N-terminal and transmembrane domain of mouse (m) MRAP1 (accession number: NM029844), zebrafish (zf) MRAP1(accession number: XP001342923.2), and rainbow trout (rt) MRAP1 (accession number: FR837908). The activation motif is highlighted in red, residues in the proposed extended activation site are underlined, and residues in the proposed reverse topology motif are highlighted in green. Identical residues in the Transmembrane Domain (TM) are highlighted in bold. **(B)** Comparison of mMRAP1, elephant shark (es) MRAP1 (accession number: XM_007903550.1), and whale shark (ws) MRAP (accession number: XM_020520012.1). The activation motif of mMRAP1 is highlighted in red. Identical residues in the proposed reverse topology motif are highlighted in green. Identical residue in the TM domain are highlighted in bold.

**Figure 4:**
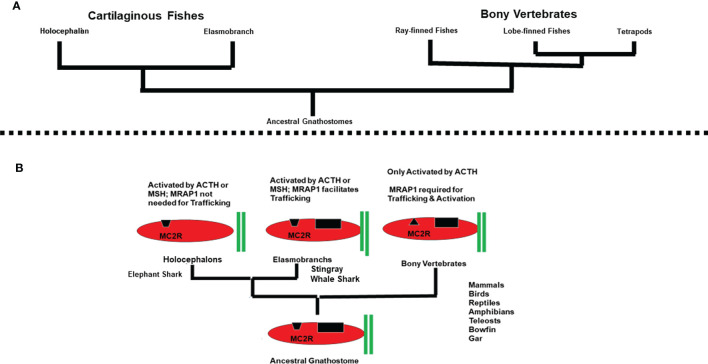
**(A)** Phylogeny of the Gnathostomes. **(B)** Trends in the co-evolution of MC2R and MRAP1. In this schematic representation (not drawn to scale), MRAP1 is represented by two green bars. Elephant shark MC2R is depicted as having an open HFRW binding site (black trapezoid). Elasmobranch MC2Rs are depicted as having an open HFRW binding site (black trapezoid) and a K(R)KRR binding site (black rectangle) for ACTH. Bony vertebrate MC2Rs are depicted as having a closed HFRW binding site (black triangle) and a K(R)KRR binding site (black rectangle) for ACTH. The presence of a proposed K(R)KRR binding site in elasmobranch MC2R orthologs and bony vertebrate MC2R orthologs is based on previous ACTH analog studies (7, 37).

During the same period, corresponding comparative structure/function studies on bony vertebrate MRAP1 orthologs have been limited to a single study on teleost MRAP1 orthologs from the zebrafish (zf; *Danio rerio*) and the rainbow trout [rt; *Oncorhynchus mykiss;* (36)]. As shown in **Figure 3A**, zfMRAP1 and rtMRAP1 have what appears to be a reverse topology motif similar to mMRAP1, and there is high primary sequence similarity in the transmembrane domains of mMRAP1, zfMRAP1, and rtMRAP1 (**Figure 3A**). In addition, zfMRAP1 and rtMRAP1 both have a four amino acid motif that corresponds to the activation motif of mMRAP1 [i.e., hydrophobic amino acid (δ)/Aspartic Acid (D)/Tyrosine (Y)/hydrophobic amino acid (δ)]. Utilizing the alanine substitution paradigm used by Sebag and Hinkle (14), replacement of the putative activation motif of rtMRAP1 (Y^10^D^11^Y^12^I^13^) with an alanine at every position completely blocked the activation of rtMC2R (3514dition, single alanine substitution of Y^10^, D^11^, Y^12^ yielded single alanine mutants of rtMRAP2 each with greatly diminished capacity to facilitate the activation of rtMC2R. However, the single alanine mutant I^13^/A^13^ had no negative effect on rtMC2R activation. Alanine substitution of residues D^16^ and Y^17^ of zfMRAP1 (**Figure 4A**) also significantly decrease activation. In the proposed activation motif of rtMRAP1 more residues appear to play a role in the activation of rtMC2R as compared to the activation motif of mMRAP1 where only alanine substitution at the Y^20^ residue resulted in a significant drop in activation (14). Whether this is a trend in non-mammalian MRAP1 orthologs, or a unique feature of rtMRAP1 has not been determined. In addition, analyses on the conserved YY motif in the N-terminal of non-mammalian bony vertebrate MRAP1 orthologs have not been done. Hence, whether there are phylogenetic trends in activation motifs of non-mammalian MRAP1 orthologs remains to be determined. However, it is clear at this time that the activation of bony vertebrate MC2R orthologs is dependent on the formation of a heterodimer with MRAP1, and these MC2R orthologs are exclusively selective for activation by ACTH perhaps as a result of the formation of the heterodimer.

## MC2R and MRAP1 Orthologs in Cartilaginous Fish

Based on the assumption that the MC2R/MRAP1 interaction originated with the ancestral gnathostomes, melanocortin receptor paralogs and mrap1 orthologs should be present in the genomes of extant cartilaginous fishes. As expected, all five melanocortin receptor paralogs have been detected in the genomes of both representative holocephalan and representative elasmobranch cartilaginous fishes (**Figure 4A**).

The first pharmacological analysis of a cartilaginous fish MC2R ortholog was done for the holocephalan, *Callorhinchus milii* [elephant shark (es); (38)], and the observations from this study did not fit neatly into the scheme for MC2R/MRAP1 interactions that was being elucidated for the bony vertebrates. For example, the esMC2R ortholog could be functionally expressed in CHO cells without co-expression with an endogenous MRAP1. In addition, the esMC2R ortholog could be activated by either ACTH or ACTH(1-13)NH_2_, the nonacetylated form of αMSH. These observations led to the speculation that the *mrap1* gene might not be present in cartilaginous fishes. However, a subsequent update of the elephant shark genome data base revealed a gene that appears to be the *mrap1* ortholog for the elephant shark (39). As shown in **Figure 3B**, the N-terminal and transmembrane domains of esMRAP1 have some features in common with mMRAP1. There is what appears to be a reverse topology motif in the N-terminal domain, and the transmembrane domain does have primary sequence similarity with mMRAP1 (**Figure 3B**). However, at the site where the activation motif should be located, rather than the expected δ-D-Y-δ motif, the putative esMRAP1 has the sequence, EYEV. In addition, co-expression of esMC2R with the putative esMRAP1 had no effect, positive or negative, on the trafficking of esMC2R to the plasma membrane, yet this co-expression did provide a modest 10-fold increase in sensitivity for stimulation by ACTH (40).

An indication that esMRAP1 did have at least some of the properties associated with bony vertebrate MRAP1 orthologs came from studies on the MC2R ortholog of the elasmobranch, *Dasyatis akajei* [stingray (sr); (41, 42)]. In the initial study of the stingray, all melanocortin receptors were cloned from the stingray genome, but no *mrap1* cDNA was detected. Expression of srMC2R in CHO cells indicted that the receptor could traffic to the plasma membrane in the absence of MRAP1. In addition, esMC2R did respond to stimulation by both ACTH and ACTH(1-13)NH_2_ but only at non-physiological concentrations of the ligands [i.e., 10^-7^M/10^-6^M; (41)]. However, co-expression of srMC2R and esMRAP1 in CHO cells resulted in a 1000-fold increase in sensitivity to stimulation by ACTH (42). This observation indicated that srMC2R needs MRAP1 for functional expression at physiological concentrations of ACTH. A later study indicated that co-expression of srMC2R and esMRAP1 significantly increased the trafficking of srMC2R to the plasma membrane of CHO cells (43). Hence, esMRAP1 has at least one of the functionalities associated with bony vertebrate MRAP1 orthologs; it can facilitate the trafficking of an MC2R ortholog to the plasma membrane. The irony of the elephant shark is that esMC2R does not require this particular property of esMRAP1.

The distinct pharmacological properties of esMC2R and srMC2R in terms of interaction with MRAP1 and ligand sensitivity presented a variation from the strict behavior of bony vertebrate MC2R orthologs and raised the question of whether there are distinct holocephalan and elasmobranch MC2R functional paradigms. To partially address this question recent studies have focused on the pharmacological properties of MC2R and MRAP1 from another elasmobranch, the whale shark [ws; *Rhincodon typus:* (43)]. When expressed alone in CHO cells, wsMC2R did traffic to the plasma membrane, and could be stimulated by either ACTH or ACTH(1-13)NH_2_; hence the response to generic melanocortin ligands appears to be a feature common to cartilaginous fish MC2R orthologs. However, under these conditions, wsMC2R could only be stimulated at non-physiological concentrations of the ligand; a feature that may be common to all elasmobranch MC2R orthologs as well. When wsMC2R and wsMRAP1 were co-expressed in CHO cells, there was a 10,000-fold increase in sensitivity to stimulation by ACTH (43). In addition, Cell Surface ELISA analysis indicated that co-transfection with wsMRAP1 significantly increased the number of receptors trafficking to the plasma membrane (43).

The primary sequence of the N-terminal domain and the transmembrane domain of wsMRAP1 is presented in **Figure 3B**. Like esMRAP1, this accessory protein has what appears to be a reverse topology motif in the N-terminal domain, and the transmembrane domain has primary sequence identity with the transmembrane domains of esMRAP1 and mMRAP1. Once again, the putative activation motif (ELDI) does not have the expected δ-D-Y-δ motif common to bony vertebrate MRAP1 orthologs. However, this motif in wsMRAP1 is nearly identical to the corresponding motif in esMRAP1.

Overall, current studies on cartilaginous fishes reveal a few novel features in the co-evolution of MC2R and MRAP1. The MC2R orthologs for the elephant shark, stingray, and whale shark can be activated by either ACTH or the smaller MSH peptides; a feature not seen in any bony vertebrate MC2R ortholog. Both wsMC2R and srMC2R require co-expression with MRAP1 to facilitate trafficking to the plasma membrane, a feature that may be common to all elasmobranch MC2R orthologs and a feature shared with all bony vertebrate MC2R orthologs. The apparent outlier in these analyses is esMC2R. The elephant shark MC2R ortholog does not require co-expression with MRAP1 for trafficking to the plasma membrane. Whether the properties of esMC2R are common to all holocephalans or unique to the elephant shark remains to be determined.

## Observations, Assumptions, and Conclusions

**Figure 4B** provides a summary of the array of MC2R/MRAP1 interactions that have currently been detected during the radiation of the gnathostomes. Three variations in the interaction between MC2R and MRAP1 are apparent. In addition, following the divergence of the ancestral cartilaginous fishes and the ancestral bony fishes from a common ancestral gnathostome (44), there was a clear dichotomy in ligand selectivity for MC2R orthologs. To account for these trends, operating assumptions on the evolution of melanocortin signaling networks in chordates are needed.

The emergence of melanocortin signaling networks is the result of the co-evolution of the melanocortin peptides and their corresponding GPCR-related MCRs. The melanocortin peptides are derived from the *pomc* gene, which is a member of the *opioid/orphanin* gene family (5). Phylogenetic analyses suggest that this gene family may have emerged in the ancestral chordates, and accordingly ACTH-like and MSH-like peptides have been detected in extant agnathans such as the lamprey, and in representatives of every major class of the gnathostomes [for reviews see (6) and (45)]. As indicated in **Figure 2**, the *mcr* gene family also appears to have emerged in the ancestral chordates (2). Based on the ligand selectivity of the majority of the melanocortin receptor subtypes (i.e., MC1R, MC3R, MC4R, MC5R), an initial speculation would be that the ancestral MCR present in the ancestral chordates could be activated by either ACTH or MSH-sized ligands. This feature was most likely retained by the MCRs that resulted from the R1 genome duplication event that is proposed to have occurred in ancestral agnathans, and the MCR paralogs that emerged as a result of the R2 genome duplication event that may have pre-dated the emergence of the ancestral gnathostomes. Based on these assumptions, it would be reasonable to propose that the MC2R ortholog of the ancestral gnathostomes (**Figure 4B**) probably could be activated by either ACTH or MSH-sized ligands. The MC2R orthologs of extant cartilaginous fishes have retained this proposed ancestral trait. Conversely, the exclusive ligand selectivity of bony vertebrate MC2R orthologs for ACTH (**Figure 4B**) can be viewed as a derived trait that emerged after the divergence of the ancestral cartilaginous fishes and ancestral bony fishes (44).

Since *mrap* genes are found in both extant cartilaginous fishes and extant bony vertebrates, some form of MC2R/MRAP1 interaction may have occurred in the ancestral gnathostomes. Thus, one interpretation of **Figure 4B** would be to hypothesize that the MC2R/MRAP1 interaction in the ancestral gnathostomes had no effect on trafficking, and perhaps a minor effect on ligand sensitivity. These traits are observed for the elephant shark MC2R/MRAP1 interaction, and in this scenario the elephant shark MC2R/MRAP1 interaction could be viewed as an ancestral trait. If the latter assumption is correct, then the trafficking function observed for the elasmobranch MC2R/MRAP1 interaction (**Figure 4B**), and the trafficking function observed for the bony vertebrate MC2R/MRAP1 interaction evolved independently and the primary sequence similarities in the TM domains of cartilaginous fish and bony vertebrate MRAP1s is the result of convergence.

An alternative and perhaps more parsimonious hypothesis to explain **Figure 4B** is to propose that the ancestral function of MRAP1 was to facilitate the trafficking of MC2R from the ER to plasma membrane in the ancestral gnathostomes. In this scenario the high degree of primary sequence similarity in the TM domains of cartilaginous fish MRAP1 orthologs and bony vertebrate MRAP1 orthologs (**Figure 3**) is viewed as an ancestral trait. The lack of a trafficking role for elephant shark MRAP1 can be viewed as a derived trait that was the result of unique point mutations in elephant shark MC2R. It appears the eMC2R has reverted to a functional state more similar to the other melanocortin receptors (i.e., MC1R, MC3R, MC4R, and MC5R) that are all MRAP1-independent. The primary sequence similarities of the TM domains of esMRAP1 relative to the other MRAP1 sequences presented in **Figure 3** point to changes in esMC2R as the potential reason for why esMC2R is now MRAP1-independent with respect to trafficking.

The divergent ligand selectivity properties of cartilaginous fish and bony vertebrate MC2R orthologs may also be a function of the primary sequence differences between these orthologs which have presumably affected the 3-dimensional shape of the respective MC2R ortholog (39). The latter study observed that the primary sequence identity between elephant shark MC2R and various bony vertebrate MC2R orthologs is only 33%; whereas, a similar comparison of MC5R orthologs indicated primary sequence identity for these orthologs of 55%. Apparently vertebrate MC2R orthologs have been diverging at a greater rate than most of the other vertebrate MCR paralogs (39). In addition, elephant shark MC2R and human MC2R represent two extremes with respect to activation by melanocortin ligands [i.e., esMC2R can be activated by either ACTH or αMSH with near equal efficacy (40); whereas hMC2R can only be activated by ACTH (1)].

The unique ligand selectivity of the mammalian “ACTH” receptor (i.e., MC2R) was reported in the 1970’s (7). In this review article two motifs in ACTH were identified that are required for the activation of the “ACTH” receptor. The primary sequence of hACTH(1-39) is SYSMEHFRWGKPVGKKRRPVKVYPNGAEDESAEAFPLEF. The “message” motif, HFRW, is found in all melanocortin peptides (4), and all MCRs have an HFRW binding site (1). Note, that the first thirteen amino acids of ACTH are the sequence for αMSH. In addition, ACTH also has the an “address motif”, KKRR, that is only found in ACTH, but not in any of the MSH-sized melanocortin peptides (for review see 5). Activation of, for example, hMC2R requires that both motifs within ACTH make contact with the receptor. However, before hMC2R can be activated, the receptor must form a heterodimer with MRAP1 to be in its functional conformation (11, 12). Presumably all other bony vertebrate MC2R orthologs should have a HFRW binding site and a KKRR binding site and require heterodimer formation to be functional. Since αMSH lacks the KKRR motif, this ligand cannot effectively activate hMC2R or other bony vertebrate MC2R orthologs at physiological concentrations of the ligand.

On the other hand, esMC2R can be activated by either ACTH or MSH-sized ligands (36) without co-expression with MRAP1. In addition, esMC2R could be fully activated with an analog of hACTH(1-24) in which the KKRR motif was replaced with alanines (37). However, this same alanine-substituted hACTH(1-24) analog could not activate hMC2R at physiologically relevant concentrations (i.e., 10^-10^M, 10^-9^M or 10^-8^M; 32). It would appear then, that hMC2R has binding sites for the HFRW motif of ACTH and the KKRR motif of ACTH; whereas, esMC2R appears to have only a binding site for the HFRW motif of ACTH.

The inability of αMSH to activate hMC2R is perplexing. An alignment of the primary sequences of esMC2R and hMC2R (39) indicated that the critical residues associated with the HFRW binding site [see (46)] in transmembrane domains 2,3 and 6 are all present in both MC2R orthologs, yet the two receptors react to stimulation by αMSH in striking different ways. Clearly the 3-dimensional structures of esMC2R and hMC2R must differ in some fundamental way. Since the crystal structure of both rhodopsin (47) and human MC4R (48) have been determined, it should be possible to model the 3-dimension structure of esMC2R. This analysis would be informative since esMC2R and hMC4R both respond to stimulation by ACTH and αMSH in essentially the same manner. However, modeling hMC2R, at present, will be more challenging. Since hMC2R, and for that matter any bony vertebrate MC2R ortholog, expressed alone in mammalian cell lines are nonfunctional (3, 12), modeling the structure of, for example, hMC2R will require x-ray crystallographic analysis of the hMC2R/hMRAP1 heterodimer. Perhaps more attainable goals in the short term would be to identify the contact site between the activation motif in the N-terminus of MRAP1 and the extracellular domain on MC2R that is involved in activating the receptor (17), and to identify the transmembrane domain on MC2R that makes contact with the transmembrane domain of MRAP1 to facilitate trafficking (13).

With respect to MRAP1, additional studies are needed on non-mammalian MRAP1 orthologs to further elucidate which residues in the δDYδ activation motif are essential for activation, and whether additional residues N-terminal to the δDYδ motif play a role in the activation process. For the cartilaginous fish MRAP1 orthologs the role of residues in the presumed activation motif of esMRAP1 (i.e., EYEV) and wsMRAP1 (i.e., ELDI) need to be evaluated. Although the first MRAP was characterized nearly sixteen years ago (10), a number of issues about these novel single pass transmembrane proteins remain unresolved.

To date over 800 G protein-coupled receptors (GPCR) have been discovered in vertebrate genomes, and within this superfamily only 30 GPCRs require interaction with an accessory protein (49). However, the significance of these accessory proteins cannot be overstated. For example, the absence of MRAP1 interferes with the functionally of the Hypothalamus/Pituitary/Adrenal Axis in humans, a critical axis for modulating homeostasis (10), and the absence of, the RAMP accessory protein renders the Angiotensin receptor, a critical receptor for blood pressure regulation, inoperative (16). Hence, understanding the interaction between these accessory proteins and their receptive GPCRs has physiological relevance. Although MRAPs and RAMPs are relatively short, single pass transmembrane proteins, there does not appear to be either primary sequence analyses or synteny analyses that would place these accessory proteins into a common gene family. Hence, it appears that these GPCR/accessory protein relationships have arisen *de novo*, which begs the question of what the “accessory proteins” were doing in cells prior to forming an interaction with their respective GPCR, and what forces brought the receptor and accessory protein together.

## Data Availability Statement

The original contributions presented in the study are included in the article/supplementary material. Further inquiries can be directed to the corresponding author.

## Author Contributions

RD and EC shared the writing of this manuscript. RD provided the outline for the subsections, and took the lead in writing sections 2, 3, and 4. EC took the lead in writing sections 1 and 5. All authors contributed to the article and approved the submitted version.

## Conflict of Interest

The authors declare that the research was conducted in the absence of any commercial or financial relationships that could be construed as a potential conflict of interest.

## Publisher’s Note

All claims expressed in this article are solely those of the authors and do not necessarily represent those of their affiliated organizations, or those of the publisher, the editors and the reviewers. Any product that may be evaluated in this article, or claim that may be made by its manufacturer, is not guaranteed or endorsed by the publisher.
